# A New Method for Predicting Erosion Damage of Suddenly Contracted Pipe Impacted by Particle Cluster via CFD-DEM

**DOI:** 10.3390/ma11101858

**Published:** 2018-09-28

**Authors:** Jiarui Cheng, Yihua Dou, Ningsheng Zhang, Zhen Li, Zhiguo Wang

**Affiliations:** 1 State Key Laboratory of Multiphase Flow in Power Engineering, Xi’an Jiaotong University, Xi’an 710049, China; nszhang@xsyu.edu.cn; 2 Department of Mechanical Engineering, Xi’an Shiyou University, Xi’an 710065, China; cjr88112@stu.xjtu.edu.cn (Y.D.); lizhen@xsyu.edu.cn (Z.L.); zhgwang@xsyu.edu.cn (Z.W.)

**Keywords:** particle cluster erosion, CFD-DEM coupling approach, particle collision interference, momentum attenuation, particle collision form

## Abstract

A numerical study on the erosion of particle clusters in an abrupt pipe was conducted by means of the combined computational fluid dynamics (CFD) and discrete element methods (DEM). Furthermore, a particle-wall extrusion model and a criterion for judging particle collision interference were developed to classify and calculate the erosion rate caused by different interparticle collision mechanisms in a cluster. Meanwhile, a full-scale pipe flow experiment was conducted to confirm the effect of a particle cluster on the erosion rate and to verify the calculated results. The reducing wall was made of super 13Cr stainless steel materials and the round ceramsite as an impact particle was 0.65 mm in diameter and 1850 kg/m^3^ in density. The results included an erosion depth, particle-wall contact parameters, and a velocity decay rate of colliding particles along the radial direction at the target surface. Subsequently, the effect of interparticle collision mechanisms on particle cluster erosion was discussed. The calculated results demonstrate that collision interference between particles during one cluster impact was more likely to appear on the surface with large particle impact angles. This collision process between the rebounded particles and the following particles not only consumed the kinetic energy but also changed the impact angle of the following particles.

## 1. Introduction

Erosion prediction is usually carried out using metal walls to evaluate the safety of process equipment, such as sudden contraction pipes. During prediction, numerous parameters, such as fluid flow characteristics, particle characteristics, particle impingement information, and target surface properties, jointly affect the efficiency and accuracy of erosion calculations, which have been widely studied and have accounted for a large number of prediction models [1]. However, almost all of these models are based on parameters or statistics of individual particles and the erosion behavior of particle clusters has received limited attention. When a particle cluster impacts the wall, the particles that initially make contact with the wall bounce back and collide with subsequent particles in a phenomenon called collision interference. This oncoming collision can decrease the impact energy and change the impact angle of the following particles, thereby causing a large error in erosion prediction. 

Erosion prediction in complex geometries adopts the computational fluid dynamics (CFD) method. CFD generally consists of three steps: Flow modeling, particle tracking, and erosion calculation. A flow field calculation is typically solved based on Navier–Stokes equations and a set of conditions, such as transient or steady state, compressible or incompressible, and laminar or turbulent flow. When the flow field is obtained, the next step in the erosion calculation is to the determine particle motion. The Eulerian or Lagrangian approach, which is based on Newton’s second law, is often used to trace particles and obtain impact parameters [2,3]. The Eulerian approach treats a particle as a continuous phase that can be deformed, broken, and bonded. However, single-particle impact momentum on the wall may be difficult to obtain using the Eulerian-Eulerian method because the pseudo-fluid element of the solid phase is easily deformed. For the Lagrangian method [4], particles are considered as an independent phase interacting with a fluid. The position and motion of particles are often obtained by solving the Equation of particle motion and particle–liquid force. In recent years, the discrete element model (DEM) [5] has received considerable interest for predicting structural erosion. DEM takes into account the normal extrusion deformation, tangential slip, and rotation of each collision particle in solving the motion equation by using a hard-sphere or a soft-sphere model. Meanwhile, the particle-scale erosion model performs a crucially important role in erosion prediction. 

When the particle impact position and parameters are derived, certain erosion models, including theoretical and empirical models, can be linked for impact calculation [6]. Classic theoretical models, such as Bitter’s [7] or Finnie’s [8] model, contain specific material characteristic parameters except for the particle impact parameters. The use of these material characteristic parameters in the predicted model improves the accuracy of the calculation for a single-particle impact. However, the predicted results for a wall that suffers from repeated particle impingement may exhibit a large error owing to metal surface fatigue and hardening. Therefore, the empirical model is used based on specific test data and applied in the calculation of special structures [9,10,11,12,13]. The advantage of the empirical model is its strong programmability and regressiveness in commercial software applications. However, each set of empirical parameter values can only be used for a specific material. Except for theoretical and empirical models, a finite element method (FEM), explicit dynamic simulation, can be employed to investigate the abrasive erosion process [14]. The FEM method can not only simulate the extrusion deformation of plastic metal when particles impact the target but can also simulate the crack propagation of brittle materials [15,16]. However, the FEM calculation process is more complicated than DEM in considering particle motion and collision, especially for the calculation of a dense phase.

Malka [17] investigated the wall erosion distribution in a special pipe section and the results showed that the erosion rate suddenly increased in the pipe contraction section. In the present work, a CFD-DEM coupling method was used to calculate particle-particle collision and particle-wall impact in a sudden contraction section, in which particles collect into a cluster. A time criterion for collision probability of a rebound particle was adopted to determine the interference of particles by coupling with a semi-empirical erosion model for cluster impact. In addition, with the momentum transfer and dissipation of the particles during a collision under consideration, the impact velocity of the particles was updated. The results showed the distribution of particle impact velocity, impact angle, erosion depth, particle-wall contact time, and the decay coefficient along the radial direction of the reducing wall. Lastly, the difference in the predictive value due to different particle collision patterns was discussed.

## 2. Solution Methodology

DEM takes into account the normal deformation, tangential slip, and rotation of each collision particle in solving the motion equation by using a hard-sphere or a soft-sphere model. In this study, we present the governing equations of fluid flow and particle motion; discuss the determination of particle interference for near-wall particle motion and provide a brief description of the erosion model.

### 2.1. Particle-Particle Interaction

The first step for getting the evolution in time of the (translational and rotational) velocity and position of the particles is to calculate the particle translation and rotational motions according to Newton’s second law [18]. The total force and torque acting on a solid particle can be expressed by [19].

(1) $$m\frac{dv}{dt}=\sum_{j=1}^{N}F_{ij}+F_{l,i}+F_{g}+F_{b}$$

(2) $$I\frac{d\omega }{dt}=\sum_{j=1}^{N}\left(T_{t,ij}+T_{r,ij}\right)$$

The total force acting on a particle is calculated as a sum of the total contact force (***F****_ij_*), fluid interaction force (***F****_l,i_*), gravitational force (***F****_g_*) and buoyancy (***F****_b_*). The total contact force is split into two components: Normal (***F****_n,ij_*) and tangential (***F****_t,ij_*). The total torque ***T****_i_* results from a vector summation of the torque at each particle-particle contact [20]. 

**Figure 1:**
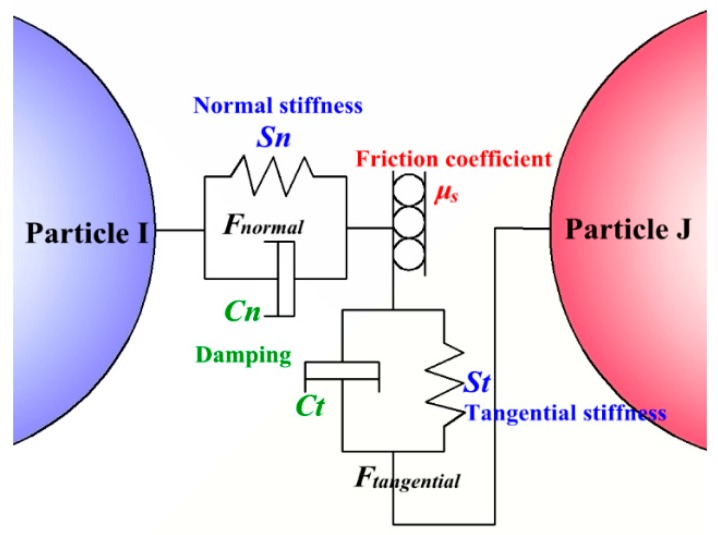
Graphic representation of the linear-spring/dashpot soft-sphere model.

For particle-particle contact, we adopted the Hertz-Mindlin (no slip) contact model to calculate the inter-particle contact force and damping force. A possible contact model in the system is given in Figure 1. Each force and moment can be treated as a spring or a damper and then, the model can be described by four elastic-damping equations. In this elastic-damping model, the normal force component is based on Hertzian contact theory and the tangential force model is based on the work of Mindlin-Deresiewicz [21]. This model has been used for contact parameter estimation for the collision of iron ore balls [22], the collision of non-spherical particles in a vibrating bed [23], and collision of particles in liquid-solid flow [6]. The results showed that this model has great calculation precision and is applicable to the calculation of particle collisions in a liquid. The total force between two particles, which contain a contact force and damping force, can be expressed by

(3) $$F_{n,ij}+F_{t,ij}=F_{n}+F_{n}^{d}+\min\left\{\mu_{s}F_{n,ij},F_{t}+F_{t}^{d}\right\}$$

where the tangential contact force (***F****_t_*) is limited by Coulomb’s law of friction and meets the relationship $F_{t}\leq -\mu_{s}\left|F_{n,ij}\right|\delta_{t}/\left|\delta_{t}\right|$ in a system without sliding [18,22]. Therefore, the normal and tangential force based on the normal and tangential overlap can be represented as $-S_{n}\delta_{n}$ and $-S_{t}\delta_{t}$, respectively. The normal and tangential damping forces are described in Tsuji’s research [24] and can be expressed as $C_{n}v_{n}^{rel}$ and $C_{t}v_{t}^{rel}$. Therefore, the total force is given by

(4) $$F_{n,ij}+F_{t,ij}=-S_{n}\delta_{n}+C_{n}v_{n}^{rel}+S_{t}\delta_{t}+C_{t}v_{t}^{rel}$$

where the stiffness constants (*S_n_* and *S_t_*), damping coefficients (*C_n_* and *C_t_*), and other parameters are defined in Table 1. In addition to the contact force between the particles, there may be some non-contact forces between the particles, which can determine the particle movement state. Van der Waals forces, capillary forces and electrostatic forces are the major non-contact forces between the particles. For particles with a micrometer or nanometer diameter, Van der Waals forces may have a great effect on the movement of the particles but the effect on the particles (*d_p_* > 0.6 mm) in this study was ignored [25]. The reason for neglecting the other forces won’t be discussed here owing to the limited space.

After we set the numerous parameters in the DEM model, the particle collision system can be well-established without considering the fluid action. However, for the particle clusters movement in an actual structure, fluid force actions on the particles play a key role in particle motion and collision. The fluid force ***F****_l,i_* often includes the drag force (***F****_d_*), virtual mass force (***F****_v_*), pressure gradient force (***F****_p_*), Saffman lift force (***F****_s_*), Basset force (***F****_ba_*), and Magnus lift force (***F****_m_*), respectively. The drag force plays a major role in the force acting on the particles by a fluid, which has been proven by literature [26]. The drag force model can be expressed by

(5) $${\vec{F}}_{d}=\frac{1}{2}C_{d}\rho_{l}A_{p}\left|u-v\right|\left(u-v\right)$$

where the following equation for the drag coefficient ($C_{d}$) is obtained

(6) $$C_{d}=\{\begin{array}{c} 24/Re_{p}\\ {\left(0.63+4.8/Re_{p}^{0.5}\right)}^{2} \end{array}\begin{array}{c} Re_{p}\leq 1\\ Re_{p}>1 \end{array}$$

where the particle Reynolds number *Re_p_* is defined as

(7) $$Re_{p}=\frac{\rho_{l}\left|u-v\right|d_{p}}{\mu }$$

In the Lagrangian description, the Saffman lift force and Magnus lift force are not considered because the particle diameter was close to 0.6 mm rather than sub-micrometer. In addition, the pressure varied very slightly over a distance of one particle diameter due to the reasonably small particles. For this reason, we neglected the pressure gradient force. Virtual mass force is not considered due to the small relative velocity between the viscous fluid and particles.

Particle collisions involve energy absorption and dissipation through momentum exchange, friction and impact restitution. It is an important factor when calculating the erosion rate using the energy method. 

### 2.2. Momentum Exchange and Energy Dissipation

**Figure 2:**
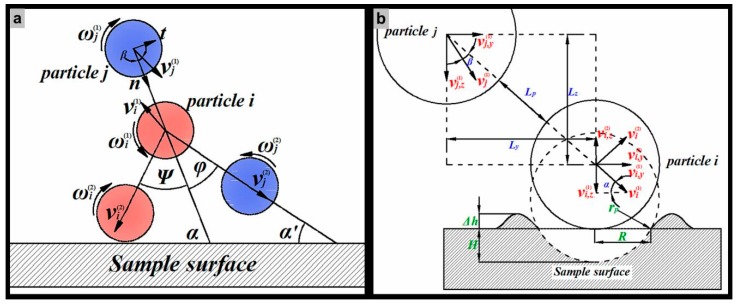
The sketch of impingement between a particle and the wall, (**a**) the particle-particle collision process; (**b**) the particle-wall contact process.

Figure 2a shows the process of collision between two particles. The subscripts (1) and (2) denote the pre-collisional and post-collisional velocities, respectively. The difference in momentum (v) and angular momentum (ω) between two instants of time is equal to the impulsive force acting on the particle, which can be expressed by [27]

(8a) $$m(v^{\left(1\right)}-v^{\left(2\right)})=J$$

(8b) $$I(\omega^{\left(1\right)}-\omega^{\left(2\right)})=-R\times J$$

Equation (8a,b) are not sufficient to determine the relationship between the pre- and post-collisional velocities. Auxiliary equations, which contain the restitution coefficient or friction coefficient, are necessary to close a set of equations. Walton [28] has proposed a hard sphere analogy of the soft-sphere model, which can be used if only two particles are in contact at one time. Jenkins [29] followed Walton and distinguished between two kinds of binary collisions: Sliding and sticking collisions. A nearly elastic collision was studied by using the constant coefficient of normal restitution and the tangential restitution to relate the translational and rotational velocities. Here we assume that all particles have the same geometry and physical properties and ignore the liquid forces. The momentum exchange between two particles can be expressed as

(9a) $$m\left(v_{in}^{\left(2\right)}-v_{jn}^{\left(2\right)}\right)\cdot r=-e_{n}m\left(v_{in}^{\left(1\right)}-v_{jn}^{\left(1\right)}\right)\cdot r\;\mathrm{for}\;\mathrm{the}\;\mathrm{normal}\;\mathrm{direction},\;\mathrm{or}$$

(9b) $$m\left(v_{it}^{\left(2\right)}-v_{jt}^{\left(2\right)}\right)\cdot t=-e_{t}m\left(v_{it}^{\left(1\right)}-v_{jt}^{\left(1\right)}\right)\cdot t\;\mathrm{for}\;\mathrm{the}\;\tan\mathrm{gential}\;\mathrm{direction}.$$

When the relative velocity of the contact points is substituted into the formula, the condition then becomes

(10a) $$v_{n}^{rel(2)}\cdot r=-e_{n}v_{n}^{rel(1)}\cdot r$$

(10b) $$v_{t}^{rel(2)}\cdot t=-e_{t}v_{t}^{rel(1)}\cdot t$$

According to Walton’s research, the post-collisional translational velocities are

(11a) $$v_{i}^{\left(2\right)}=v_{i}^{\left(1\right)}-\frac{1}{2}\left(1+e_{n}\right)\left(\Delta v\cdot r\right)r-\frac{K}{2}\frac{1+e_{t}}{1+K}v_{i}^{rel(1)}$$

(11b) $$v_{j}^{\left(2\right)}=v_{j}^{\left(1\right)}-\frac{1}{2}\left(1+e_{n}\right)\left(\Delta v\cdot r\right)r-\frac{K}{2}\frac{1+e_{t}}{1+K}v_{j}^{rel(1)}$$

and the rotational velocities are

(12a) $$\omega_{i}^{\left(2\right)}=\omega_{i}^{\left(1\right)}-\frac{1+e_{t}}{R(1+K)}r\times v_{i}^{rel(1)}$$

(12b) $$\omega_{j}^{\left(2\right)}=\omega_{j}^{\left(1\right)}-\frac{1+e_{t}}{R(1+K)}r\times v_{j}^{rel(1)}$$

where the expressions of parameter ∆*v*, *v^rel^* and *K* are shown in Table 1. There is an angle between ***v*** and ***r*** that can determine whether a collision is sliding or sticking. When this angle is greater than or equal to a critical angle θ*, a sliding collision occurs. Otherwise, a sticking collision takes place. The tangential coefficient of restitution can be expressed by [30]

(13) $$e_{t}=-1+\mu \left(1+e_{n}\right)\left(1+\frac{md_{p}^{2}}{4I}\right)\cot\theta^{*}$$

This hard sphere analogy of the soft-sphere collision dynamics greatly reduces the computational time for a soft-sphere model. When the particle cluster impacts on the wall, many particles will bounce back from the wall and collide with others. Therefore, the kinetic energy of particle *j* may be reduced by the collision of particle *i* before it impacts the wall. We define the ratio of pre-collisional velocity to post-collisional velocity as the decay coefficient, which includes the normal momentum decay coefficient (*λ_n_*), the tangential momentum decay coefficient (*λ_t_*) and the energy decay coefficient (*λ_e_*). The following definitions have been used in general.

(14) $$\mathrm{Normal}\;\mathrm{decay}\;\mathrm{coefficient}\;\lambda_{n}=v_{j}^{\left(2\right)}/v_{j}^{\left(1\right)}=1-\left[\frac{1}{2}\left(1+e_{n}\right)\left(\Delta v\cdot r\right)r-\frac{K}{2}\frac{1+e_{t}}{1+K}v_{j}^{rel(1)}\right]/v_{j}^{\left(1\right)}\;\mathrm{Tan}\mathrm{gential}\;\mathrm{decay}\;\mathrm{coefficient}\;\lambda_{t}=\omega_{j}^{\left(2\right)}/\omega_{j}^{\left(1\right)}=1-\left[\frac{1+e_{t}}{R(1+K)}r\times v_{j}^{rel(1)}\right]/\omega_{j}^{\left(1\right)}\;$$

When the particle-particle collision is considered as a nearly elastic one, according to the law of conservation of energy, the exchange of kinetic energy between two particles can be expressed by the following relation

(15) $$\frac{1}{2}mv_{j}^{\left(1\right)}+\frac{1}{2}mv_{i}^{\left(1\right)}=\frac{1}{2}mv_{j}^{\left(2\right)}+\frac{1}{2}mv_{i}^{\left(2\right)}+\Delta E_{n}+\Delta E_{t}$$

where the change of translational and rotational kinetic energy have the forms of [18]

(16) $$\Delta E_{n}=-\frac{1}{2}m\left(1-e_{n}\right){\left(r\cdot \Delta v\right)}^{2}-m\frac{1+e_{t}}{7}\left(t\cdot v^{rel}\right)\left(t\cdot \Delta v\right)+m{\left(\frac{1+e_{t}}{7}\right)}^{2}{\left(t\cdot v^{rel}\right)}^{2}\;\Delta E_{t}=m\frac{1+e_{t}}{14}d_{p}\left|r\times v^{rel}\right|\left(r\times t\right)\left(\omega_{i}^{\left(1\right)}+\omega_{j}^{\left(1\right)}\right)+\frac{5}{2}m{\left(\frac{1+e_{t}}{7}\right)}^{2}{\left(r\times v^{rel}\right)}^{2}\;$$

If the energy exchange is expressed in the form of a reduced velocity, i.e., 12mp(Δv’)2=ΔEn+ΔEt, the decrease in velocity of particle *j* can be expressed by an energy decay coefficient, which is

(17) $$\lambda_{e}=\frac{v_{j}^{\left(1\right)}-\Delta v^{\prime }}{v_{j}^{\left(1\right)}}=1-\sqrt{\frac{2(\Delta E_{n}+\Delta E_{t})}{m{\left(v_{j}^{\left(1\right)}\right)}^{2}}}$$

### 2.3. Particle-Wall Interaction

In order to determine whether there is a collision between two particles near the wall, the particle-wall contact is taken into account as shown in Figure 2b. Unlike particle-particle contact models, particles are treated as fully rigid bodies when they come into contact with the wall. Comparing the rebound time, *t*_1_, for particle *i* and the moving time *t*_2_ for particle *j*, if *t*_1_ > *t*_2_, the particle *j* will collide with particle *i* before it leaves the wall region, on the contrary, it will not. The rebound process for particle *i* consists of two steps including a contact step and a release step. Therefore, the rebound time can be expressed as *t*_1_ = *t_e_* + *t_r_*. When the effect of elastic deformation is ignored, the depth of the extruding lips is given by

(18) $$\Delta h=H-r^{2}/\left(2r_{p}\right)$$

The strain caused by the normal stress of indentation can be calculated by

(19) $$\varepsilon =\frac{\Delta h}{H}=\frac{H-r^{2}/\left(2r_{p}\right)}{H}$$

According to Hooke’s law, the average force acting on a metal surface during the indentation process is

(20) $$F_{m}=E\int_{0}^{R}2\pi r\left(\frac{H-r^{2}/\left(2r_{p}\right)}{H}\right)dr=E\pi r_{p}H$$

where the maximum depth of indentation, H, can be described as [31]

(21) $$H={\left(\frac{m_{p}{\left(v_{i,z}^{\left(1\right)}\right)}^{2}}{\pi d_{p}P_{z}}\right)}^{1/2}$$

According to the mutual principle of forces, the normal force for a single particle is equal to the force of a metal surface. When the indentation depth reaches a maximum, the particle’s normal velocity, ***v****_i,z_*, closes to 0 m/s. Therefore, the contact time with the metal surface, based on the theorems of particle momentum, can be expressed by the following equation.

(22) $$t_{e}=\frac{m_{p}v_{i,z}^{\left(1\right)}}{F_{m}}=\frac{m_{p}v_{i,z}^{\left(1\right)}}{E\pi r_{p}H}$$

In the process of contact between the particle *i* and the wall, the moving distance of the particle *j* along the centerline is

(23) $$L_{p,e}=t_{e}\cdot v_{j}^{\left(1\right)}\cdot \cos\beta =\frac{m_{p}v_{i,z}^{\left(1\right)}v_{j}^{\left(1\right)}\cos\beta }{E\pi r_{p}H}$$

According to Habib’s research [32,33], particles move toward the centerline of the pipe as they approach the sudden contraction section. As shown in Figure 2b, the movement of two particles can be treated as a meeting problem in the Z direction as well as a catching-up problem in the Y direction after the rebound of particle *i*. Therefore, the release time (*t_r_*) should meet the following relationship in order to satisfy the time adaptive condition of particle collision.

(24) $$t_{r,y}\in [L_{y}/(v_{j,y}^{\left(1\right)}-v_{i,y}^{\left(2\right)}),\left(L_{y}+d_{p}\right)/(v_{j,y}^{\left(1\right)}-v_{i,y}^{\left(2\right)})]\;t_{r,z}\in [L_{z}/(v_{j,z}^{\left(1\right)}+v_{i,z}^{\left(2\right)}),\left(L_{z}+d_{p}\right)/(v_{j,z}^{\left(1\right)}+v_{i,z}^{\left(2\right)})]\;$$

If the distance between the two particles is far greater than the particle diameter and if the acceleration of particles is ignored, the critical inter-particle distance in the release process will be given by

(25) $$L_{p,r}=\sqrt{{\left(L_{y}-d_{p}\right)}^{2}+{\left(L_{z}-d_{p}\right)}^{2}}$$

where

$$L_{y}/(v_{j,y}^{\left(1\right)}-v_{i,y}^{\left(2\right)})=L_{z}/(v_{j,z}^{\left(1\right)}+v_{i,z}^{\left(2\right)})$$

Then, the critical inter-particle distance in the process of a particle-particle collision near the wall can be expressed by

(26) $$L_{p}=L_{p,r}+L_{p,e}=\sqrt{{\left(L_{y}-d_{p}\right)}^{2}+{\left(L_{z}-d_{p}\right)}^{2}}+\frac{m_{p}v_{i,z}^{\left(1\right)}v_{j}^{\left(1\right)}\cos\beta }{E\pi r_{p}H}$$

where the release velocity for particle *i* can be obtained using the velocity component based on the relation [34]

$$\begin{array}{l} v_{i,z}^{\left(2\right)}/v_{i,z}^{\left(1\right)}=0.993-1.76\alpha +1.56\alpha_{}^{2}-0.49\alpha_{}^{3}\\ v_{i,y}^{\left(2\right)}/v_{i,y}^{\left(1\right)}=0.988-1.66\alpha_{}+2.11\alpha_{}^{2}-0.67\alpha_{}^{3} \end{array}$$

The velocity loss rate for a single particle in the process of particle-wall contact is given by

(27) $$\kappa =(1-v_{i}^{\left(2\right)}/v_{i}^{\left(1\right)})\times 100\%$$

### 2.4. Governing Equations of Fluid Flow

For the liquid phase, the governing Equations comply with the law of conservation of mass and momentum in terms of local-average variables. Taking the volume fraction of the continuous phase and the particle-liquid interaction force into account, the continuous carrier liquid flow equations are based on Model A [35] and are given by

(28a) $$\frac{\partial }{\partial t}(\alpha_{l}\rho_{l})+\nabla \cdot (\alpha_{l}\rho_{l}u)=0$$

(28b) $$\frac{\partial }{\partial t}(\alpha_{l}\rho_{l}u)+\nabla \cdot (\alpha_{l}\rho_{l}uu)=-\alpha_{l}\nabla p+\nabla \cdot (\alpha_{l}\tau )+\alpha_{l}\rho_{l}g-F_{p,i}$$

where the particle force acting on the liquid (***F****_p,i_*) should be equal to the force of the liquid phase (***F****_l,i_*) acting on the solid phase in the opposite direction. In the above equations, the fluid volume fraction is obtained from the relation $\alpha_{l}1-\sum_{j=1}^{N}V_{p}/\Delta V_{l}$ and the viscous stress tensor ***τ***is defined as

(29) $$\tau =\alpha_{l}\mu \left(\nabla u+{\left(\nabla u\right)}^{T}-\frac{2}{3}\left(\nabla u\right)\delta_{k}\right)$$

In the process of the coupling calculation, the motion of a particles phase is obtained by DEM, which applies Newton’s laws of motion at the individual particle level, while the flow of continuum fluid is described by the local averaged Navier–Stokes equations that can be solved using the CFD approach.

### 2.5. Erosion Model

A detailed literature survey by Meng and Ludema [36] revealed that more than 30 erosion models exist for particle impact erosion to date. All prediction models basically include the particle impact velocity and impact angle, such as the model proposed by Chen [11], McLaury [12] and Ahlert [13]. According to Chen’s research, erosion can be calculated by using Equation (30)

(30) $$E_{R}=X\cdot E_{RA}+Y\cdot E_{RI}$$

where *X* and *Y* represent the percentage of grouped particles and independent particles in the total impact particles, respectively. Therefore, the total erosion rate is controlled by multiple impacts of different kinds of particles. The detailed erosion models for each impact are given by [11]

(31) $$E_{RA}=\sum_{i=1}^{N_{1}}KF_{s}{\left(\lambda v_{p}\right)}^{m}f\left(\alpha \right)\;\mathrm{for}\;\mathrm{clustered}\;\mathrm{particle}\;\mathrm{impacts}$$

(32) $$\;E_{RI}=\sum_{i=1}^{N-N_{1}}KF_{s}v_{p}^{m}f\left(\alpha \right)\;\mathrm{for}\;\mathrm{independent}\;\mathrm{particle}\;\mathrm{impacts}$$

where *λ = λ_n_·λ_t_·λ_e_*; *F_s_ =* 1.0 for sharp, 0.53 for semi-rounded, or 0.2 for fully rounded sand particles. The impact angle function *f*(*α*) is given by

(33) $$f\left(\alpha \right)=\left\{ \begin{array}{ll} a\alpha^{2}+b\alpha & \mathrm{for}\;\alpha \leq \theta \\ x\cos^{2}\alpha \sin\left(w\alpha \right)+y\sin^{2}\alpha +z & \mathrm{for}\;\alpha >\theta \end{array}\right.$$

The erosion properties of the target wall material (super 13Cr stainless steel) have been studied by a jet flow system at the Xi’an Shiyou University [37] and at a critical impact angle, *θ =* 70°. The new erosion sub-model parameters used in the erosion calculation are shown in Table 2. In the post-processor, the erosion was visualized as the local wall thickness loss rate on different radial positions of the reducing wall.

## 3. Implementation and Verification

### 3.1. Numerical Solving Step

**Figure 3:**
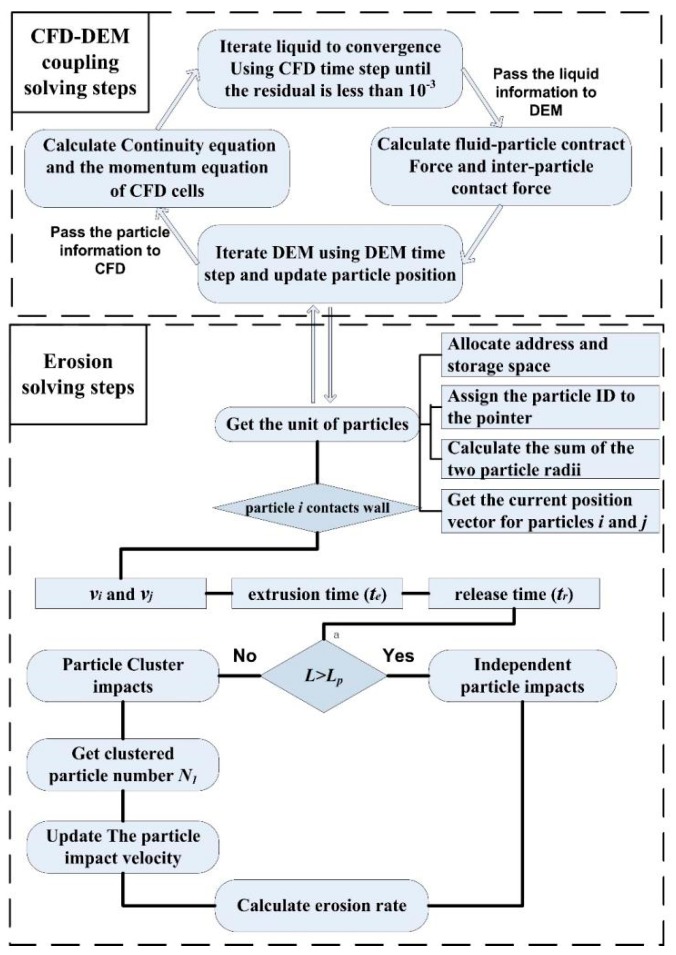
The erosion simulation scheme.

In the current study, erosion prediction involved the calculation of liquid–particle, particle-particle, and particle-wall interactions, which were completed via CFD coupled with DEM. The CFD code ANSYS FLUENT [38] was employed to model the continuous phase flow. Meanwhile, discrete particle motion modeling was accomplished using the DEM code EDEM [39] as shown in Figure 3. The governing equations (Equation (28a,b)) were discretized in a finite volume form and then, the discretized equations were solved using a semi-implicit method for pressure linked equations (SIMPLE), which is described by Ferziger [40] in detail. The explicit time integration method was used to calculate the motion of the particles. When a stable fluid region was obtained, the drag and contract forces between the fluid and particle were calculated using the DEM time step. Subsequently, new particle positions were transferred to the coupling module between CFD and DEM. The flow field was updated until the results satisfied the accuracy requirement. Two calculations were performed independently but were coupled at regular intervals, commonly with multiple DEM time steps for a single CFD time step. 

In the coupling process, space and time steps of CFD and DEM must satisfy a certain correspondence. Although two phases were created in Fluent using the Eulerian-Eulerian model, conservation equations for the solid phase were not solved. Therefore, it was necessary to calculate the volume fraction of the CFD mesh cell occupied by the particles. In order to obtain the position of the particles before and after the collision, the CFD mesh cell occupied by the particles was usually divided into a number of small spaces. The particle volume fraction within a particular mesh cell was, therefore, the percentage of the points as given by

$$\alpha_{p}=1-\alpha_{l}=\sum_{i=1}^{N}\frac{N_{p}V_{p}}{N_{t}}$$

The grid size, *∆x*, which can be expressed by *∆x* = (*V_p_*/*N_p_*)^1/3^, determined the number of CFD points within the mesh cell of a particle. It was usually necessary to ensure that *N_p_* ≧ 1 and *N_p_* < *N*_l_. Meanwhile, a grid size that was too small would increase the amount of calculation and a grid size that was too large size may ignore collision details. Therefore, the grid size was set to 0.2 mm for the CFD mesh in this work.

In general, the CFD calculation time size and the DEM calculation time size must satisfy the following relationship: Firstly, the minimum time step size needed to satisfy the CFD calculation convergence condition. Secondly, the ratio of the DEM time step to the Rayleigh time step should be in the range of 5–30%. Thirdly, the ratio of the CFD time step to the DEM time step must be an integer. The Rayleigh time step between two particles is given by [41]

(34) $$\Delta t_{Ra}=\frac{\pi r_{p}\sqrt{\rho_{p}/G}}{0.163\nu +0.877}$$

Studies have shown that the DEM time step is proportional to the Rayleigh time step and the ratio is within the range of 0.1–0.5 [42,43,44]. Here, the DEM time step was equal to one-fifth of the Rayleigh time step, that is, Δ*t_D_* = 0.2 × ∆*t_Ra_* and the shear modulus can be expressed as *G* = *Y*/2(2 + *ν*)(1 − *ν*). In order to obtain the contact parameters between the particles and the wall, the DEM time step needs less than the contact time (Equation (22)). Therefore, the time step was set to be 2 × 10^−6^ s for DEM and 4 × 10^−5^ s for CFD.

When the particles move closer to the reducing wall, they will change direction and accelerate under the action of the liquid force [32,33], resulting in an increase in the distance between the particles. Therefore, whether the particles in a cluster collide with each other near the target and thus affect the erosion prediction is determined by the following steps:

**Calculation of the critical interparticle distance:** The distance between adjacent particles was calculated when the front particle made contact with the wall. If *L* < *L_p_*, the subsequent impact on the wall was treated as a clustered particle impact; otherwise, the impact was considered as an independent particle impact.

**Acquisition of clustered particles**: The distance determination method was used to divide the form of particle impingement on the wall and impact parameters were calculated by classification.

**Determination of the decay coefficient:** For the clustered particles, the next step was to obtain the decay coefficient *λ* and the new impact angle for each colliding particle after a particle-particle collision.

### 3.2. Boundary Conditions

**Figure 4:**
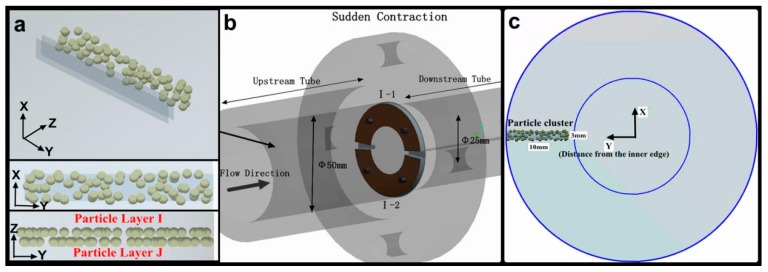
Structure of the contraction section and the super 13Cr samples, (**a**) the distribution and arrangement of the particles in two particle layers; (**b**) the diagram of a sudden contraction section; (**c**) the location of a particle cluster.

The detailed parameters for fluid, particle, and geometry are summarized in Table 3. As shown in Figure 4, the particles were generated on two layers (Particle Layers *I* and *J*) of virtual quadrilateral planes, with the two planes radially distributed and spaced from each other by 0.05 mm. Each virtual plane was10 mm in length, 3 mm in width, perpendicular to the pipe axis and close to the pipe wall. One of the purposes of this setting was to induce the maximum number of particles to impact the reducing wall because only particles near the wall of the upstream pipe can impact the wall. Another purpose was to distribute the particles as far as possible in the radial direction of the target wall.

Particle Layer *I*, which was in front of Particle Layer *J*, represented particles that initially impacted the wall and were not subject to particle collisions. By contrast, the particles in Layer *J* may suffer from particle-particle collision interference near the wall. In each particle layer, particles were randomly positioned, thus satisfying the multiple possibilities of particles impacting the wall. Therefore, the number of particles mentioned in Table 3 is an average value and may change with different particle-producing positions. Meanwhile, a particle-moving calculation at each velocity was repeated 10 times to count the particle impact parameters on different radial walls because not all surfaces in the calculation process were impacted. Moreover, the inducing wall was considered to be under a no-slip condition, where *u_r_*|*_r_* = *u_x_*|*_r_*_= 0_ = 0 and *әu_r_*/*әx* = 0 and a fully developed incompressible flow was considered at the velocity inlet and exit sections.

### 3.3. Full-Scale Experiment

**Figure 5:**
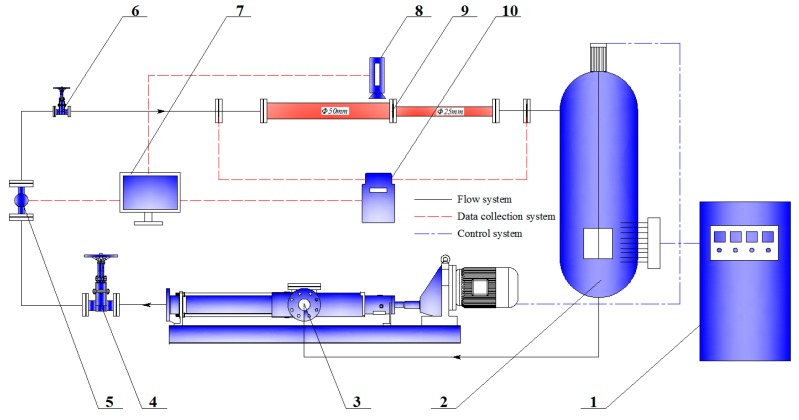
Schematic diagram of the experimental set-up: (1) Electrical control cabinet; (2) liquid storage tank containing the electric heater (2 m^3^); (3) screw pump; (4) (6) gate valve; (5) flow meter; (7) computer; (8) high-speed camera; (9) test section; (10) pressure transducer.

A full-scale pipe flow experiment was used to study particle clusters during erosion and to verify the simulation results. As shown in Figure 5, the experimental loop mainly comprised a contraction section, a screw pump (with a flow range of 5–60 m^3^/h), an electric heating agitator, a temperature and pressure sensor, a magnetic flowmeter (8712HR, Emerson, Rosemount. Co., Boulder, CO， USA), four gate values, a control cabinet, a computer, and connecting pipes. After the test fluid containing the particles was mixed, stirring and electric heating units were turned on until the temperature reached the required value and then the pump was turned on. The gate valve in the test pipe was opened when the flow reached stability and the related data, including a particle motion image, flow pressure, and temperature were recorded.

**Figure 6:**
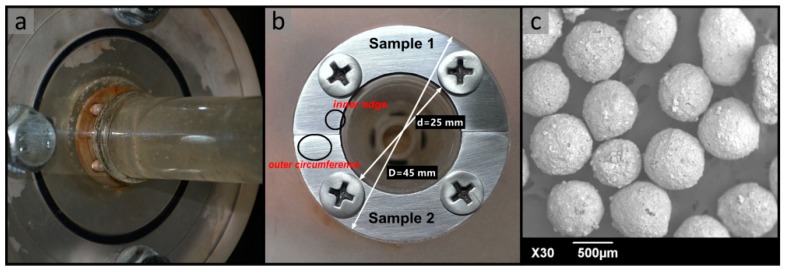
Physical map of the test section: (**a**) Diagram of the contraction section; (**b**) installation drawing of the samples; (**c**) particle surface morphology scanned by SEM.

The test section (Figure 6a) consisted of a large-diameter pipe (*D* = 50 mm), a small-diameter pipe (*D* = 25 mm), and two annular samples (super 13Cr stainless steel), which were fixed on the reducing wall by four screws (Figure 6b). The reducing wall was divided into two parts according to the particle impact frequency. The inner edge of the sample surface withstood multiple overlapping impacts with the formation of craters, platelets, and extruding lips. The outer circumference surface mainly suffered from an independent particle impingement. The test fluid was added with 0.4 wt.% hydroxypropyl guar gum as a thickener and 0.3 wt.% inorganic boron as a cross-linking agent. The viscosities of the liquid before and after crosslinking were 26 and 375 mPa. Round ceramsite, with a density of 1850 kg/m^3^ and an average diameter of 0.65 mm, was used as an erosion abrasive (Figure 6c). The mass concentration of the particles in the suspension was 50 kg/m^3^. The flow velocities of the experimental liquid were set to 1.5, 2.5, and 3.5 m/s, respectively. The exposed surface was sealed with epoxy resin and ground using SiC emery paper grade 1200 prior to installation. A MEMRECAM SP-614 high-speed camera (NAC. Co., Ltd., Tokyo, Japan) was used to observe the particle clusters with a spatial resolution of 0.01 μm and the sample surface profiles were verified using an H1200WIDE confocal scanning laser microscope (Lasertec. Co., Ltd., Tokyo, Japan) with a scanning rate of 120 fps.

**Figure 7:**
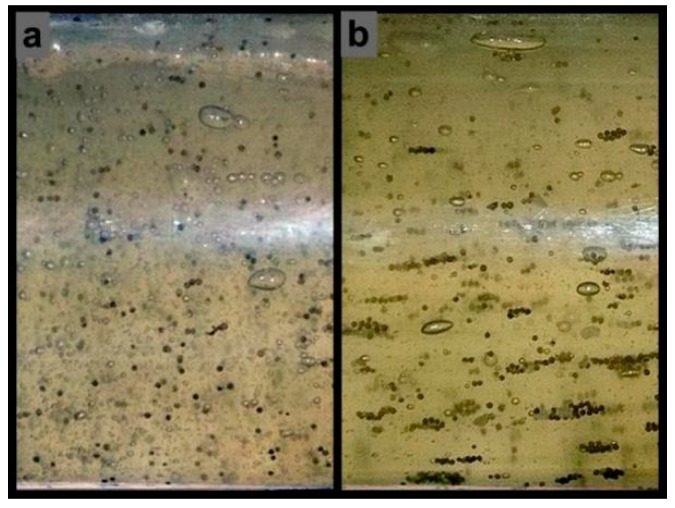
Distribution of particles in different fluids, (**a**) dispersed particles in the base fluid; (**b**) particle clusters in the crosslinking fluid.

**Figure 8:**
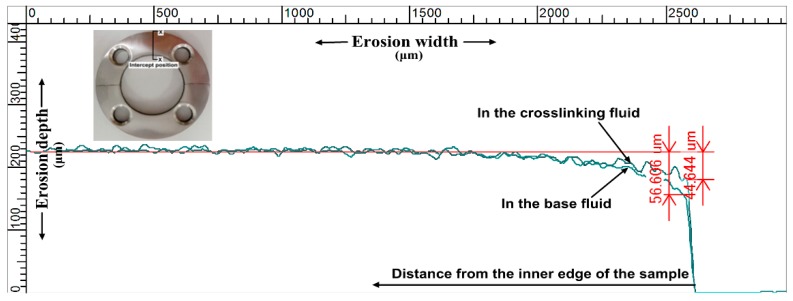
The comparison of the erosion depth between the particles in the base fluid and the crosslinking fluid.

Figure 7 shows the particle distribution in the upstream pipe at a flow velocity of 2.5 m/s. Most of the particles were uniformly suspended in the base fluid (Figure 7a), resulting in an independent impact erosion (IIE) of the particles on the reducing wall. However, in the crosslinking fluid (Figure 7b), numerous particles collected into clusters, possibly leading to cluster impact erosion. Measurements of profiles of the erosive surface at the same flow velocity in the base fluid and crosslinking fluid are shown in Figure 8. The results show notably different erosion depths on the inner edge surface and the erosion difference gradually decreased along the radial direction. The maximum differences in erosion depth were 18.37, 11.96, and 8.43 μm, at the flow velocities of 1.5, 2.5, and 3.5 m/s, respectively. By ignoring the effects of metal corrosion, fatigue damage, and surface hardening, we identified that the critical cause of the erosion difference was particle collision interference, which diminished the impact energy of a fraction of the particles on the wall. As shown by the measurement results, the effect of particle interference was magnified by more particle impacts on the inner edge of the sample, whereas extremely small effects were observed in areas that were rarely impacted by particles (for a detailed description of the division of the erosion area on the reducing wall, we refer the reader to our previous work [45]).

## 4. Results

**Figure 9:**
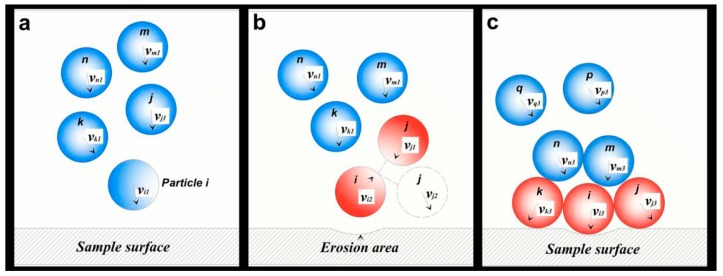
Schematic diagrams of the particle-wall impact and particle-particle collision in a cluster, (**a**) distribution of the particles in pipe flow; (**b**) initial contact between the front particle and the wall; (**c**) the rebound of the front particle; (**d**) mutual interference of the particles.

As shown in Figure 7b, most of the particles formed many particle clusters and gathered together across the upstream section. When the particle cluster impacted the wall, the particle-wall contact process may involve three steps: Initial contact between the front particle and the wall, rebound of the front particle, and collision interference between particles. The initial contact occurred between particle *i* and the metal surface (Figure 9a). If particles were too close, particle *j* in the cluster would collide with rebounding particle *i* (Figure 9b). Afterward, numerous subsequent particles may collide with each other and even form an unstable accumulation layer (Figure 9c).

In the current study, the impact parameters between Particle Layer I and the reducing wall were used to obtain the distribution of the erosion crater depths, the particle-wall contact time, and the particle velocity attenuation. Meanwhile, the particles in Particle Layer J were used to determine the particle interference and calculate particle cluster erosion.

### 4.1. Particle-Wall Impingement

**Figure 10:**
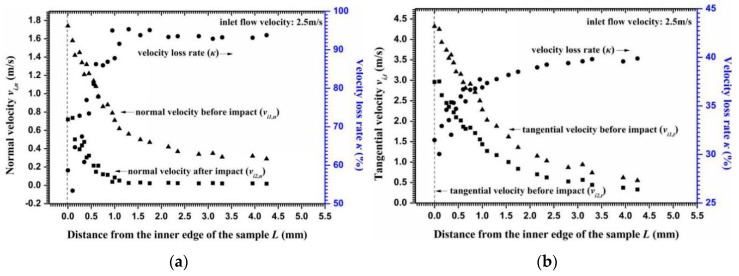
Velocity components of a particle in Particle Layer I along the radial surface. (**a**) Normal impact velocities; (**b**) Tangential impact velocities.

Target wall deformation included the extrusion and recovery processes. The time spanning all processes was dominated by the particle impact velocity and angle. Figure 10a,b show the normal and tangential velocity components before and after the particles impacted the wall, respectively. The particle tangential velocities were markedly higher than that of the normal velocities because of the steering effect of the particles [32,33], that is, the small particle impact angle increased the particle tangential velocity. According to the relationship between the velocity decay ratio and impact angle, the normal velocity loss rate changes from 60 to 90% along the radial surface (Figure 10a). Most of the impact energy was dissipated in the plastic work and elastic wave energies and the loss rate reached 90%, as confirmed by Hutchings [46,47]. Meanwhile, the tangential velocity loss rates, which were almost half of the normal velocity loss rate, approached 30% on the inner surface and 40% on the outer circumference (Figure 10b).

**Figure 11:**
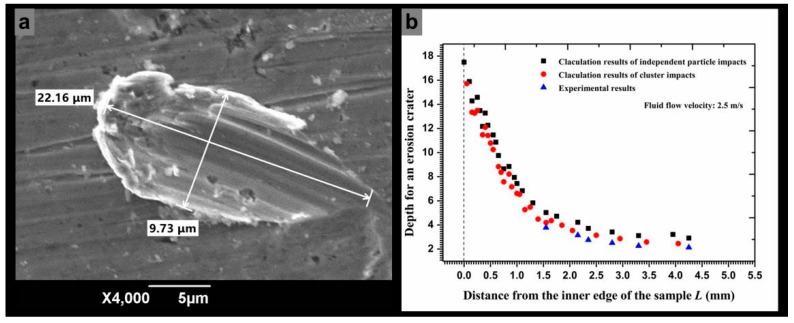
Depths for an erosion crater obtained by experimental measurement and numerical calculation, (**a**) SEM micrograph of the single impact crater on the outer circumference surface; (**b**) erosion depth caused by independent particle impacts and cluster impacts versus experimental results.

**Figure 12:**
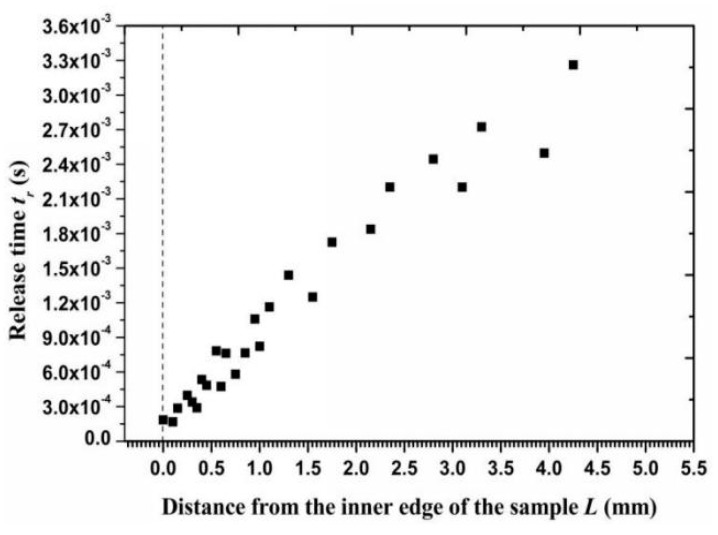
Release time of the particles after impacting on the wall versus the distance from the inner edge.

The depth of an erosion crater can be approximated using Equation (31) when the particle impact velocity and angle were obtained. However, a significant empirical coefficient *P_n_*, which indicates the average pressure between a particle and the metal surface, needs to be obtained experimentally. The geometric dimensions of the 20 similar craters (Figure 11a) on the outer circumference of a sample surface were measured to determine the coefficient value. The obtained *P_n_* was equal to 2.1 × 10^6^ Pa for super 13Cr stainless steel. Figure 11b shows the calculated erosion depth along the radial surface. The depth of an independent erosion crater gradually increased from the inner edge to the outer circumference, with maximum and minimum erosion depths of 17.8 and 2.9 μm, respectively. Hence, the calculated contact time using Equation (32) stabilized in the microsecond time scale and was notably lower than the particle release time (a difference of 1000 times) so that *t*_1_ was approximately equal to *t_r_*. Figure 12 shows the release time for Particle *I* along the radial direction. The minimum release time for a particle that impacts the inner surface was 1.8 × 10^−4^ s and the maximum release time was 3.3 × 10^−3^ s. If a particle collision occurred after particle *i* rebounded from the wall, then a lower release time indicates that a shorter interparticle distance is required to allow two adjacent particles to collide.

### 4.2. Particle Cluster Erosion

**Figure 13:**
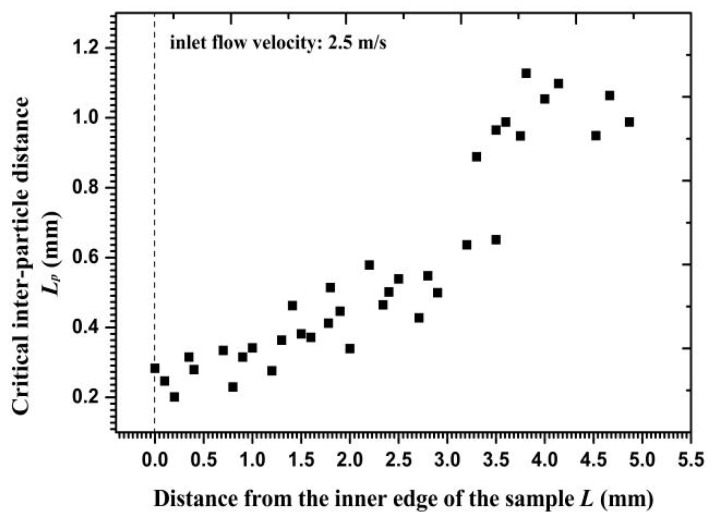
Critical inter-particle distance versus the distance from the inner edge.

**Figure 14:**
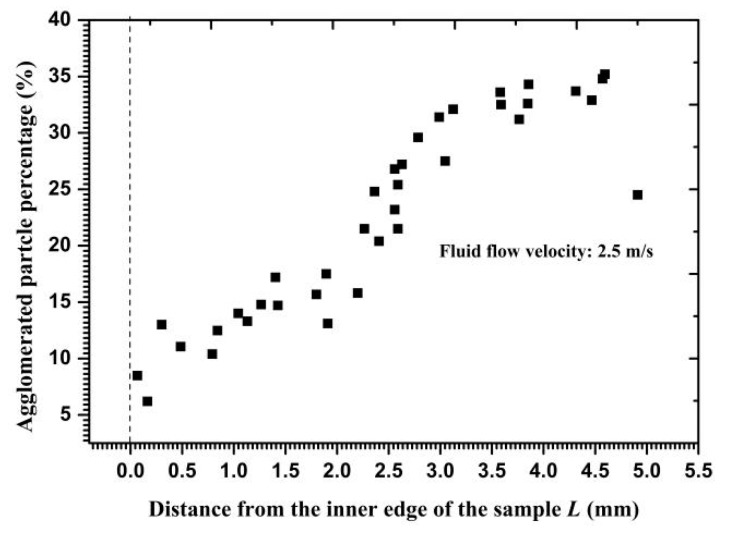
Clustered particle percentage versus the distance from the inner edge.

According to the values of particle velocity, particle release time, and the force acting on the particle, critical interparticle distances were calculated along the radial direction and are shown in Figure 13. The distances increased gradually from the inner edge to the outer circumference, reflecting that more particles may collide with each other near the outer circumference owing to a loose distance limit. By repeatedly calculating the distance between the two-layer particles when Particle *I* made contact with the wall, the clustered particle percentages of total impact particles were calculated and are exhibited in Figure 14. Calculations and statistical results showed that only 10% of the total impacted particles on the inner edge and 35% on the outer circumference were affected by collision interference before impacting the wall. Therefore, the particles impacting on the wall as a cluster were less than half of the total number of particles. Meanwhile, particles that were closer to the inner edge showed more independent particle impacts, thereby demonstrating the diminished effect of a high velocity gradient on clustered particle collision.

**Figure 15:**
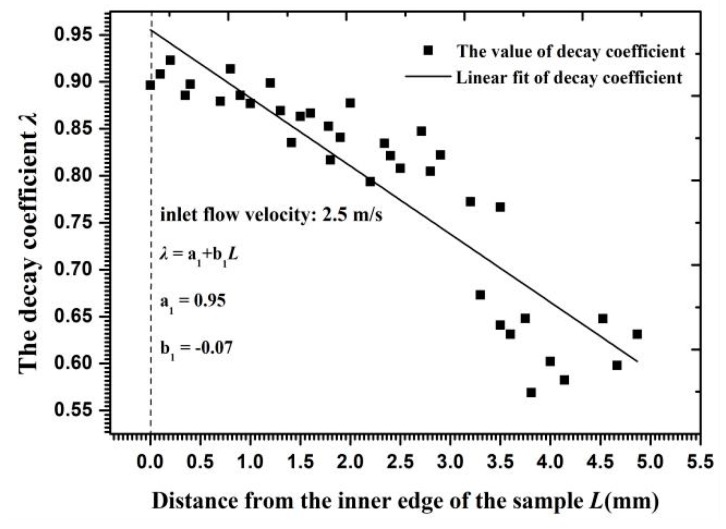
The velocity decay coefficient in collision versus the distance from the inner edge.

**Figure 16:**
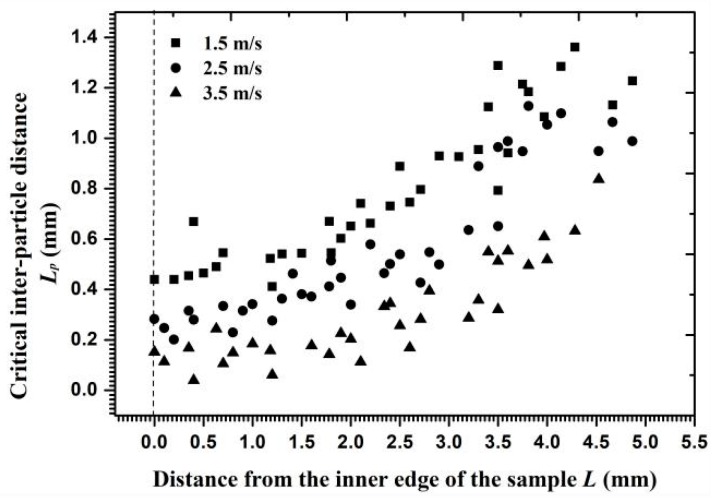
The critical inter-particle distance versus the distance from the inner edge at different flow velocities.

**Figure 17:**
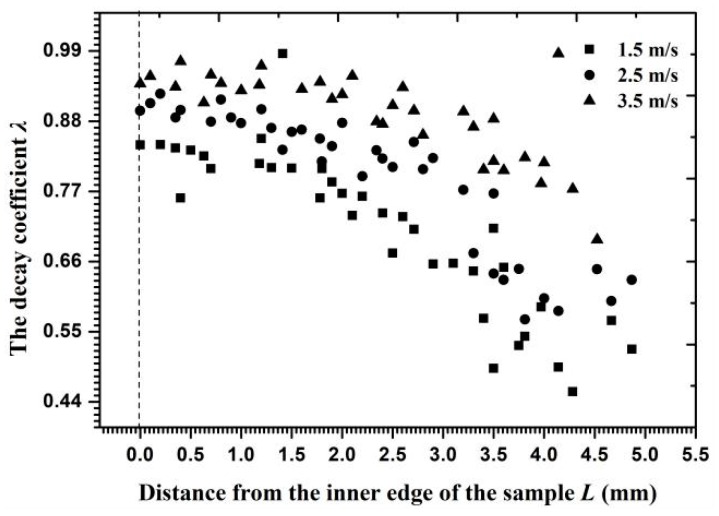
The velocity decay coefficient versus the distance from the inner edge at different flow velocities.

Figure 15 shows the velocity decay coefficient for each particle in Layer *J* along the radial surface. The coefficient values approached 0.9 on the inner edge and 0.55–0.7 on the outer circumference, indicating that the kinetic energy consumption of Particle *J* on the outer circumference exceeded that on the inner edge. When the inlet flow velocity was altered, the velocity gradient and the angle of velocity component near the reducing wall partly changed and then the critical interparticle distance reached a new range. Figure 16 shows that the critical interparticle distance decreased with increasing flow velocity, reflecting that particles must be closer to each other for a collision to occur at a high flow velocity. In addition to its influence on the critical interparticle distance, an increasing flow velocity changed the decay coefficient of the particle velocity, as shown in Figure 17. Owing to the growth of the opposite collision velocity between two particles with an increasing flow velocity, the decay coefficient increased on average by 11% and approached 1 with an increased flow velocity by 1 m/s. Therefore, the effect of a cluster impact on the erosion calculation at a high flow velocity was less than that at a low flow velocity on the reducing wall. 

**Figure 18:**
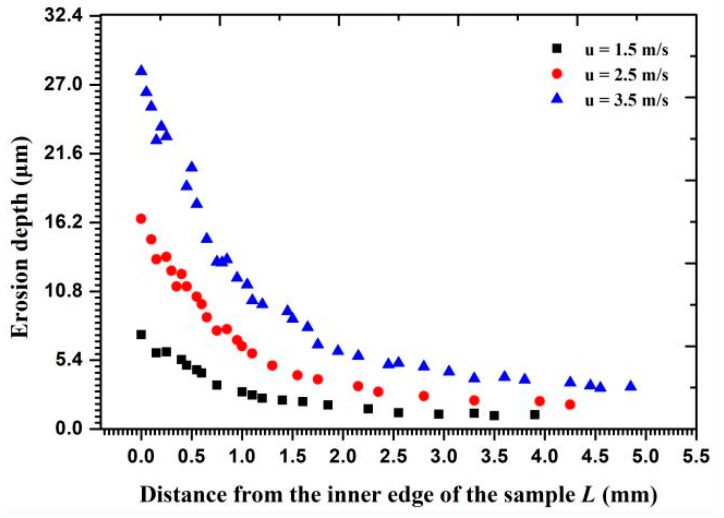
Metal erosion depth under particle cluster impacts at different flow velocities.

The calculated results of the erosion depth by particle cluster and by independent particle impact in the radial direction are shown in Figure 11b for comparison. Compared with the calculated results of erosion depth caused by an independent particle impact, the erosion depths caused by particle cluster were also determined in the radial direction. The value of the cluster impact erosion depth approximates that of the independent particle impact on the inner edge but shows a significant difference on the outer circumference. With a change in the fluid flow velocity, as shown in Figure 18, the main erosion position was on the inner surface and the erosion depth markedly increased on the inner edge with an increasing flow velocity. More particles impacted the wall at a high flow velocity because the particles possessed a higher inertial force to hit the wall head-on. Moreover, the particle impact areas also increased with the increased flow velocity.

### 4.3. Discussion

**Figure 19:**
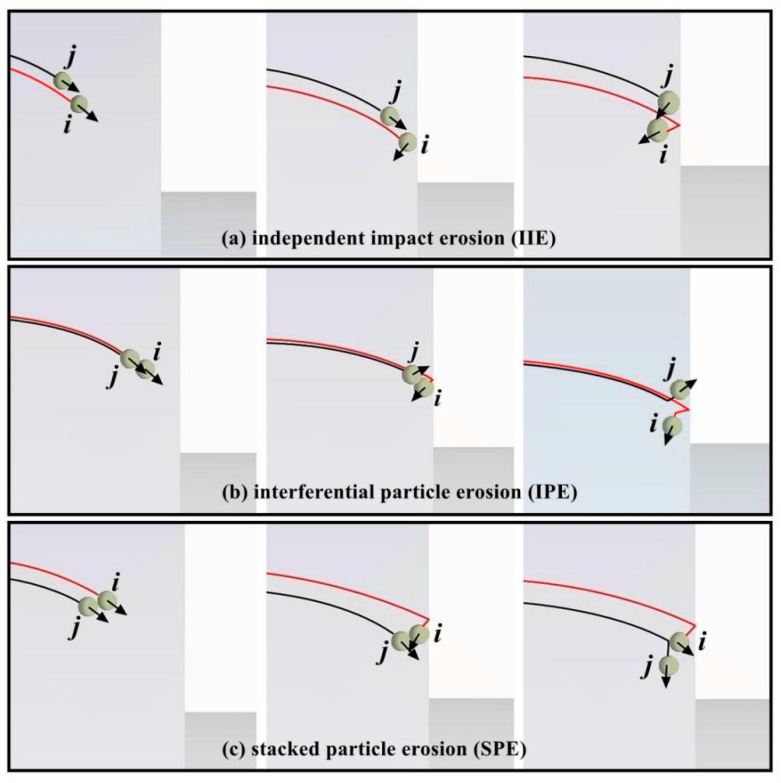
Schematic diagrams of particle-wall and particle-particle collisions between two particles.

The above results exhibit the effect of particle collision prior to impacting a wall on erosion and the following chapters discuss the changes in particle impact velocity under different particle collision forms. Based on the trajectory and collision form of two particles, the clustered particle erosion can be divided into three patterns, including IIE, interferential particle erosion (IPE), and stacked particle erosion (SPE). When the trajectories of the two particles are changed, the different particle-wall impact forms as shown in Figure 19.

**Independent Impact Erosion (IIE)**: When particle *j* moves on the outer side of particle *i* (Figure 19a), the particle *i* initially impacts the wall and then rebounds back to the other side. Meanwhile, particle *j* follows particle *i* and does not collide with the latter. The impact between the two particles and the wall is independent of each other.

**Interferential Particle Erosion (IPE)**: When the trajectories of two particles are extremely close, as shown in Figure 19b, particle *i* impacts the wall and then collides with particle *j*. Particle *j* may impact the wall again after the collision, but the new impact energy of particle *j* should be attenuated during the particle-particle collision.

**Stacked Particle Erosion (SPE)**: When the motion path of particle *j* is inside particle *i* (Figure 19c), particle *j* may be subjected to a head-on collision after particle *i* rebounds off the reducing wall. Therefore, the second impact between a particle and the wall may be attributed to particle *i* instead of particle *j*. This result causes the actual velocity in the second wall impingement to decrease twice.

**Figure 20:**
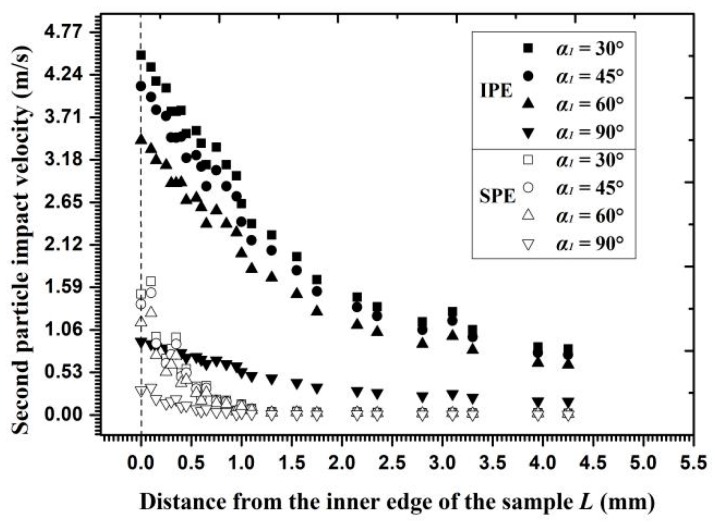
The second particle impact velocity on the wall for interferential particle erosion (IPE) and stacked particle erosion (SPE). The second impact particle is particle *j* in IPE as well as is particle *i* in SPE.

IIE has been investigated in numerous aspects and is thus no longer discussed. As for IPE and SPE, different collision positions during momentum transfer between two particles result in different scattering angles and velocity decay rates for particle *j*. According to the relationship between the scattering angle and the new impact angle, that is, *α*_1_ = *α* − *φ* (Figure 2a), the particle impact velocities in the second particle-wall impingement at different new impact angles are displayed in Figure 20. Given that the particle during the second impingement suffers twice the momentum loss in SPE and only once in IPE, the particle impact velocity in IPE is almost three times the velocity in SPE on the inner edge and almost 10 times on the outer circumference. Therefore, the effect of SPE on decreasing erosion extends far beyond the IPE and both erosion types exert more influence than IIE.

## 5. Conclusions

The erosion characteristics of a reducing wall in a sudden contraction were analyzed by CFD-DEM coupling methods. A novel identification approach of particle cluster erosion was applied in the particle-wall impact calculation and the results provide the distribution of particle impact velocity, impact angle, erosion crater depth, critical interparticle distance, and velocity decay coefficient along the radial surface. The influence of cluster collision on the erosion rate was verified by a full-scale experiment. Meanwhile, the simulation results of particle cluster erosion were compared with the experimental results with different decay velocities. The conclusions derived from the present study can be summarized as follows:

(1) Formation of particle clusters results in interparticle collisions when certain particles bounce back from the wall and the succeeding particle erosion rate decreases with the change in particle impact velocity.
(2) During particle impact and rebound, the contact time of the target surface is less than that of the particle rebound time before the particle collides with another particle. The decay rate of a normal impact velocity changes from 60 to 90% along the radial surface and the decay rate of the tangential velocity changes in the range of 30 to 40%.
(3) A smaller critical interparticle distance, which indicates that particles must be closer to each other for a collision to exist, results in a lower clustered particle percentage of the total impact particles. Therefore, the probability and frequency of a particle cluster impact on the outer circumference are larger than those on the inner edge. Meanwhile, by updating the particle impact velocity and angle, the relative error between the calculation results and the experimental results was reduced by almost 11% compared with that of the complete independent impact setting.
(4) The second impact velocity of the particle in IPE is almost three times the velocity in SPE on the inner edge and almost 10 times on the outer circumference because the particle velocity decays once in IPE and twice in SPE. The growth of flow velocity not only decreases the critical interparticle distance but also the decay coefficient in particle collision, indicating that the effect of a cluster impact on erosion can be weakened by an increasing flow velocity.

## Figures and Tables

**Table 1:** Equations for the discrete element method (DEM) model.

Parameter	Expression
Normal stiffness constant (*S_n_*)	$\frac{4}{3}E^{*}\sqrt{R^{*}\delta_{n}^{}}$
Tangential stiffness constant (*S_t_*)	$8G^{*}\sqrt{R^{*}\delta_{n}}$
Normal damping coefficient (*C_n_*)	$-2\sqrt{\frac{5}{6}}\gamma \sqrt{S_{n}m^{*}}$
Tangential damping coefficient (*C_t_*)	$-2\sqrt{\frac{5}{6}}\gamma \sqrt{S_{t}m^{*}}$
Torque by tangential forces (*T_t,ij_*)	$R_{i}\times F_{t,ij}$
Rolling friction torque (*T_r,i_*_j_)	$-\mu_{r}\left|F_{n,ij}\right|R^{*}\cdot \omega_{i}/\left|\omega_{i}\right|$
Equivalent elastic modulus (E*)	$\frac{1}{E^{*}}=\frac{1-\nu_{i}^{2}}{E_{i}}+\frac{1-\nu_{j}^{2}}{E_{j}}$
Equivalent shear modulus (G*)	$\frac{1}{G^{*}}=\frac{2(2+\nu_{i})(1-\nu_{i})}{Y_{i}}+\frac{2(2+\nu_{j})(1-\nu_{j})}{Y_{j}}$
Equivalent radius (R*)	$\frac{1}{R^{*}}=\frac{1}{R_{i}}+\frac{1}{R_{j}}$
Equivalent mass (m*)	$\frac{1}{m^{*}}=\frac{1}{m_{i}}+\frac{1}{m_{j}}$
Dimensionless coefficient (*γ*)	$\ln e/\sqrt{\ln^{2}e+\pi^{2}}$
The relative velocities of the centers of the spheres before and after a collision (∆*v*)	$\Delta v=v_{i}-v_{j}$
The relative velocity of the contact points	$v^{rel}=\Delta v+\frac{d_{p}}{2}\left(\omega_{i}+\omega_{j}\right)\times r$
The unit tangential vector (*t*)	$t=\frac{\left(v^{rel}\times r\right)\times r}{\left|v^{rel}\times r\right|}$
Dimensionless coefficient (*K*)	$K=\frac{4I}{md_{p}^{2}}$

**Table 2:** The erosion sub-model parameters used in erosion prediction.

*K*	*F_s_*	*m*	*a*	*b*	*x*	*y*	*z*	*w*	*θ*
7.8 × 10^−^^8^	0.35	1.57	5.9 × 10^−5^	−7.2 × 10^−5^	0.75	−0.21	0.83	−1.2	70

**Table 3:** CFD-DEM simulation parameters and geometric dimensions of the domain.

DEM Parameter	Value
**Liquid phase**	
Liquid density (kg/m^3^)	1020
Liquid viscosity (mPa·s)	375
**Solid phase**	
Diameter (mm)	0.65
Mass (mg)	0.26
Sphericity	0.85
Particle density (kg/m^3^)	1850
Number of particles per calculation in each layer	60 ± 5
Dimensionless coefficient *K*	0.4
Coefficient of normal restitution *e_n_*	0.95
Coefficient of normal restitution *e_r_*	0.36
Coefficient of friction *µ*	0.1
**Geometry**	
Upstream pipe length in the axial direction (mm)	400
Downstream pipe length in the axial direction (mm)	200
Upstream pipe diameter (mm)	50
Downstream pipe diameter (mm)	25
Total grid cell number	845, 732
Length of virtual plane (mm)	10
Width of virtual plane (mm)	3
The distance from virtual planes to target wall (mm)	300

## Nomenclature

*A_p_*	particle cross-sectional area, m^2^	*t_e_*	contact time, s
*C_d_*	drag coefficient, dimensionless	*t_r_*	release time, s
*C_n_*	normal damping coefficient, kg·s^−1^	*t*_1_	rebound time, s
*C_t_*	tangential damping coefficient, kg·s^−1^	*t*_2_	moving time for particle *j*, s
*D*	inside diameter of circular pipe, m	∆*t_D_*	DEM time step, s
*d_p_*	diameter of particle, m	∆*t_Ra_*	Rayleigh time step, s
*e_n_*	restitution coefficient in the normal direction, dimensionless	*T_r_*	rolling friction torque, N·m
*e_t_*	restitution coefficient in the tangential direction, dimensionless	*T_t_*	torque generated by tangential forces, N·m
*E*	elastic modulus, Pa	*u*	fluid flow velocity, m
*E_R_*	total erosion rate, kg/kg	∆*v*	the relative velocities of the centers of the spheres before and after a collision, m·s^−1^
*E_RA_*	erosion rate caused by independent particle impact, kg/kg	*v_n_*	particle normal velocity, m·s^−1^
*E_RI_*	erosion rate caused by grouped particle impact, kg/kg	*v_t_*	particle tangential velocity, m·s^−1^
*E**	equivalent elastic modulus, Pa	*v^ref^*	the relative velocity of the contact points, m·s^−1^
*∆E_n_*	normal energy loss of a particle caused by particle-particle collision, J	*V_p_*	volume of particle, m^3^
*∆E_t_*	tangential energy loss of a particle caused by particle-particle collision, J	*V_l_*	volume of liquid cell, m^3^
*F_b_*	buoyancy, N	∆*x*	space step, m
*F_d_*	drag force, N	*Y*	Young’s modulus, Pa
*F_g_*	gravitational force, N	*a*	empirical constants in angle functions, dimensionless
*F_l,i_*	the fluid force acting on a particle, N	*b*	
*F_n_*	inter-particle contact force in the normal direction, N	*w*	
*F_p,i_*	the particle force acting on the liquid, N	*x*	
*F_ij_*	contact force between particles, N	*y*	
*F_t_*	inter-particle contact force in the tangential direction, N	*z*	
*F_s_*	particle shape coefficient, dimensionless	*Greek symbols*	
*F_n_^d^*	inter-particle damping force in the normal direction, N	*ω_i_*	unit angular velocity vector of the object, rad·s^−1^
*F_t_^d^*	inter-particle damping force in the tangential direction, N	*λ*	decay coefficient in the collision, dimensionless
*G*	shear modulus, Pa	*κ*	particle velocity loss rate in process of particle-wall impact, %
*G**	equivalent shear modulus, Pa	*ν*	poisson ratio, dimensionless
*H*	maximum depth of indentation, m	*δ*	tangential displacement, m
*Δh*	depth of the extruding lips, m	*μ*	dynamic viscosity, Pa·s
*I*	moment of inertia, m^4^	*β*	The angle between the particle velocity and the centerline of the two particles, °
*J*	particle momentum, kg·m·s^−1^	*δ_k_*	identity tensor
*K*	dimensionless coefficient, dimensionless	*δ_n_*	normal overlap, m
*L*	distance from the inner edge of the sample, m	*δ_t_*	tangential overlap, m
*L_p_*	critical inter-particle distance of particle, m	*ρ_l_*	liquid density, kg·m^−3^
*L_p,e_*	the moving distance of the particle j along the centerline, m	*μ_s_*	friction coefficient, dimensionless
*L_p,r_*	the critical inter-particle distance in the release process, m	*μ_r_*	rolling friction coefficient, dimensionless
*L_y_*	particle spacing in the Y direction, m	*φ*	scattering angle for particle j, °
*L_z_*	particle spacing in the Z direction, m	*ψ*	scattering angle for particle *i*, °
*m_p_*	single particle mass, kg	*α*	particle impact angle, °
*m**	equivalent mass, kg	*α*_1_	new particle impact angle, °
*N*	total particle number, dimensionless	*α_l_*	fluid volume fraction, %
*N_p_*	the number of sample points contained within the mesh cell of the particle, dimensionless	*θ*	critical angle of particle impact, °
*N*_1_	the number of particles in the particle group, dimensionless	*θ**	critical angle between particles, °
*N_t_*	total number of sample points of the particle, dimensionless	*ε*	strain caused by the normal stress of indentation, m
*P_n_*	function of properties of the material, Pa	*Superscripts*	
*r*	normal unit vector, dimensionless	*n*	flow behavior index
*r_p_*	radius of the erosion crater, m	*m*	impact velocity power law coefficient
*R*	radius of particle, m	**(1)**	pre-collision
*R_i_*	maximum radius of deformation, m	**(2)**	post-collision
*R**	equivalent radius, m	*Subscripts*	
*R_l_*	inside radius of a circular pipe, m	*i*	particle *i*
*S_n_*	normal stiffness, N·m^−1^	*j*	particle* j*
*S_t_*	tangential stiffness, N·m^−1^	*z*	normal direction
*t*	tangential unit vector, dimensionless	*y*	tangential direction

## References

[1] Parsi M., Najmi K., Najafifard F., Hassani S., McLaury B.S., Shirazi S.A. (2014). A comprehensive review of solid particle erosion modeling for oil and gas wells and pipelines applications. J. Nat. Gas. Sci. Eng..

[2] Wood R.J.K., Jones T.F., Ganeshalingam J., Miles N.J. (2004). Comparison of predicted and experimental erosion estimates in slurry ducts. Wear.

[3] Zhu H., Han Q., Wang J., He S., Wang D. (2015). Numerical investigation of the process and flow erosion of flushing oil tank with nitrogen. Powder Technol..

[4] Njobuenwu D.O., Fairweather M. (2012). Modelling of pipe bend erosion by dilute particle suspensions. Comput. Chem. Eng..

[5] Zhu H.P., Zhou Z.Y., Yang R.Y., Yu A.B. (2007). Discrete particle simulation of particulate systems: Theoretical developments. Chem. Eng. Sci..

[6] Zhang Y., McLaury B.S., Shirazi S.A. (2009). Improvements of particle near-wall velocity and erosion predictions using a commercial CFD code. J. Fluids Eng..

[7] Bitter J.G.A. (1963). Study of erosion phenomenon–1,2. Wear.

[8] Finnie I. (1960). Erosion of surfaces by solid particles. Wear.

[9] Chen J.K., Wang Y.S., Li X.F., He R.Y., Han S., Chen Y.L. (2015). Erosion prediction of liquid-particle two-phase flow in pipeline elbows via CFD–DEM coupling method. Powder Technol..

[10] Zhao Y., Xu L., Zheng J. (2016). CFD-DEM simulation of tube erosion in a fluidized bed. AiChE J..

[11] Chen X., McLaury B.S., Shirazi S.A. (2004). Application and experimental validation of a computational fluid dynamics (CFD)-based erosion prediction model in elbows and plugged tees. Comput. Fluids.

[12] McLaury B.S. (1996). Predicting Solid Particle Erosion Resulting from Turbulent Fluctuations in Oilfield Geometries. Ph.D. Thesis.

[13] Ahlert K. (1994). Effects of Particle Impingement Angle and Surface Wetting on Solid Particle Erosion of AISI 1018 Steel. Master’s Thesis.

[14] Lv Z., Huang C.Z., Zhu H.T., Wang P.Y., Liu Z.W. (2015). FEM analysis on the abrasive erosion process in ultrasonic-assisted abrasive waterjet machining. Int. J. Adv. Manuf. Technol..

[15] Bedon C., Santarsiero M. (2018). Laminated glass beams with thick embedded connections-Numerical analysis of full-scale specimens during cracking regime. Compos. Struct..

[16] Larcher M., Arrigoni M., Bedon C., Van Doormaal J.C.A.M., Haberacker C., Husken G., Millon O., Saarenheimo A., Solomos G., Thamie L. (2016). Design of blast-loaded glazing windows and façades: A review of essential requirements towards standardization. Adv. Civ. Eng..

[17] Malka R., Nešić S., Gulino D.A. (2007). Erosion–corrosion and synergistic effects in disturbed liquid-particle flow. Wear.

[18] Blais B., Lassaigne M., Goniva C., Fradette L., Bertrand F. (2016). Development of an unresolved CFD-DEM model for the flow of viscous suspensions and its application to solid-liquid mixing. J. Comput. Phys..

[19] Iqbal N., Rauh C. (2016). Coupling of discrete element model (DEM) with computational fluid mechanics (CFD): A validation study. Appl. Math. Comput..

[20] Gupta P., Sun J., Ooi Y.J. (2016). DEM-CFD simulation of a dense fluidized bed: Wall boundary and particle size effects. Powder Technol..

[21] Mindlin R.D., Deresiewicz H. (1953). Elastic spheres in contact under varying oblique forces. J. Appl. Mech..

[22] Barrios G.K.P., Carvalho R.M.D., Kwade A., Tavares L.M. (2013). Contact parameter estimation for DEM simulation of iron ore pellet handling. Powder Technol..

[23] Chung Y.C., Liao H.H., Hsiau S.S. (2013). Convection behavior of non-spherical particles in a vibrating bed: Discrete element modeling and experimental validation. Powder Technol..

[24] Tsuji Y., Tanaka T., Ishida T. (1992). Lagrangian numerical-simulation of plug flow of cohesionless particles in a horizontal pipe. Powder Technol..

[25] Chen J., Anandarajah A. (1996). Van der Waals attraction between spherical particles. J. Colloid Interface Sci..

[26] Zhang Y. (2006). Application and Improvement of Computational Fluid Dynamics (CFD) in Solid Particle Erosion Modeling.

[27] Crowe C.T., Schwarzkopf J.D., Sommerfeld M., Tsuji Y. (2011). Particle-particle interaction. Multiphase Flows with Droplets and Particles.

[28] Walton O.R., Roco M.C. (1993). Numerical simulation of inelastic, frictional particle-particle interactions. Particulate Two-Phase Flow.

[29] Jenkins J.T., Zhang C. (2002). Kinetic theory for identical, frictional, nearly elastic spheres. Phys. Fluids.

[30] Yang L., Padding J.T., Kuipers J.A.M. (2016). Modification of kinetic theory for frictional spheres, Part I: Two-fluid model derivation and numerical implementation. Chem. Eng. Sci..

[31] Huang C.K., Chiovelli S., Minev P., Luo J., Nandakumar K. (2008). A comprehensive phenomenological model for erosion of materials in jet flow. Powder Technol..

[32] Habib M.A., Ben-Mansour R., Badr H.M., Kabir M.E. (2008). Erosion and penetration rates of a pipe protruded in a sudden contraction. Comput. Fluids.

[33] Habib M.A., Badr H.M., Ben-Mansour R., Kabir M.E. (2007). Erosion rate correlations of a pipe protruded in an abrupt pipe contraction. Int. J. Impact Eng..

[34] Grant G., Tabakoff W. (1975). Erosion prediction in turbomachinery resulting from environmental solid particles. J. Aircraft.

[35] Gidaspow D. (1994). Transport Equations. Multiphase Flow and Fluidization.

[36] Meng H.C., Ludema K.C. (1995). Wear models and predictive Equations: their form and content. Wear.

[37] Zhao Y.A., Cai W.B., Cui L., Cheng J.R., Dou Y.H. Erosion of premium connection cross-over joint in solid-liquid flow. Proceedings of the International Conference on Engineering Technology and Application ICETA2015.

[38] (2014). ANSYS FLUENT 15.0 User Guide.

[39] (2015). EDEM 2.7 User Guide.

[40] Ferziger J.H., Peric M., Leonard A. (1997). Computational methods for fluid dynamics. Phys. Today.

[41] Kremmer M., Favier J.F. (2001). A method for representing boundaries in discrete element modelling—Part II: Kinematics. Int. J. Numer. Meth. Eng..

[42] Chen X., Zhong W., Zhou X., Jin B., Sun B. (2012). CFD–DEM simulation of particle transport and deposition in pulmonary airway. Powder Technol..

[43] Hobbs A. (2009). Simulation of an aggregate dryer using coupled CFD and DEM methods. Int. J. Comput. Fluid D.

[44] Xu Y., Kafui K., Thornton C., Lian G. (2002). Effects of material properties on granular flow in a silo using DEM simulation. Part. Sci. Technol..

[45] Cheng J.R., Zhang N.S., Li Z., Dou Y.H., Cao Y.P. (2017). Erosion failure of horizontal pipe reducing wall in power-law fluid containing particles via CFD–DEM coupling method. J. Fail. Anal. Prev..

[46] Hutchings I.M., Macmillan N.H., Rickerby D.G. (1981). Further studies of the oblique impact of a hard sphere against a ductile solid. Int. J. Mech. Sci..

[47] Hutchings I.M., Winter R.E. (1974). Solid particle erosion studies using single angular particles. Wear.

